# Physiologic and histopathologic effects of targeted lung denervation in an animal model

**DOI:** 10.1152/japplphysiol.00565.2018

**Published:** 2018-10-25

**Authors:** James P. Hummel, Martin L. Mayse, Steve Dimmer, Philip J. Johnson

**Affiliations:** ^1^Division of Cardiology, University of Wisconsin, Madison, Wisconsin; ^2^Nuvaira, Inc., Minneapolis, Minnesota

**Keywords:** COPD, nonpharmacologic therapy, targeted lung denervation

## Abstract

Parasympathetic efferent innervation of the lung is the primary source of lung acetylcholine. Inhaled long-acting anticholinergics improve lung function and symptoms in patients with chronic obstructive pulmonary disease. Targeted lung denervation (TLD), a bronchoscopic procedure intended to disrupt pulmonary parasympathetic inputs, is an experimental treatment for chronic obstructive pulmonary disease. The physiologic and histologic effects of TLD have not previously been assessed. Eleven sheep and two dogs underwent circumferential ablation of the main bronchi with simultaneous balloon surface cooling using a lung denervation system (Nuvaira, Inc., Minneapolis, MN). Changes in pulmonary air flow resistance were monitored before and following TLD. Four animals were assessed for the presence or abolishment of the sensory axon-mediated Hering-Breuer reflex before and following TLD. Six sheep were histologically evaluated 30 days post-TLD for the extent of lung denervation (axonal staining) and effect on peribronchial structures near the treatment site. No adverse clinical effects were seen in any treated animals. TLD produced a ~30% reduction in pulmonary resistance and abolished the sensory-mediated Hering-Breuer reflex. Axonal staining was consistently decreased 60% at 30 days after TLD. All treated airways exhibited 100% epithelial integrity. Damage to other peribronchial structures was minimal. Tissue 1 cm proximal and distal to the treatment was normal, and the esophagus and periesophageal vagus nerve branches were unaffected. TLD treatment effectively denervates the lung while protecting the bronchial epithelium and minimizing effects on peribronchial structures.

**NEW & NOTEWORTHY** The feasibility of targeted lung denervation, a new minimally invasive therapy for obstructive lung disease, has been demonstrated in humans with preliminary clinical studies demonstrating improvement in symptoms, pulmonary function, and exercise capacity in patients with chronic obstructive pulmonary disease. This preclinical animal study demonstrates the ability of targeted lung denervation to disrupt vagal inputs to the lung and details its physiologic and histopathologic effects.

## INTRODUCTION

Autonomic innervation of the lung is provided by the vagus nerve via the pulmonary plexus, which originates at the level of the main carina and extends nerve branches along each mainstem bronchi that terminate in distal airways. These nerves control airway relaxation and constriction using integrated networks of parasympathetic preganglionic neurons in the brainstem, postganglionic neurons in the lung, and sensory neurons in the nodose and jugular ganglia. Preganglionic efferent fibers coursing through these pulmonary branches of the vagal nerve provide signals to postganglionic neurons of bronchial ganglia via acetylcholine release. Each intrinsic bronchial ganglion innervates airway smooth muscle and other lung effectors (10, 13, 29). A primary function of efferent stimulation of postganglionic neurons is to regulate smooth muscle tone and facilitate bronchoconstriction (10, 16, 31). Chronic obstructive pulmonary disease (COPD) and asthma are characterized by persistent or recurrent airflow obstruction that may be linked to overstimulation of these parasympathetic efferent fibers. The primary therapies for obstructive pulmonary disorders are intended to reduce airway resistance. Long-acting pharmacologic blockade of cholinergic nervous input (efferent input) has become a mainstay of treatment for COPD, resulting in marked improvements in dyspnea, frequency of exacerbations, and lung function (6, 30, 39).

Modifying vagal nerve input to the lung has repeatedly been shown to alter the resistance of air flow in and out of the lungs by changing airway diameter and smooth muscle tone. Surgical denervation of the lung causes decreases in bronchial smooth muscle tone and reduces resistance to airflow in the lungs of several animal models (5, 8, 9, 18, 20, 27). Conversely, stimulation of the pulmonary vagus nerves increases smooth muscle tone and increases resistance to airflow in the lungs (3, 18, 33). Similarly, the clinical application of vagotomy to treat COPD (2, 36) and severe asthma (14) has been shown to increase lung function and exercise capacity while reducing disease symptoms. Direct surgical access to the pulmonary branches of the vagus nerve, however, would require thoracotomy, rendering it clinically impractical in this high risk population.

**Fig. 1.**
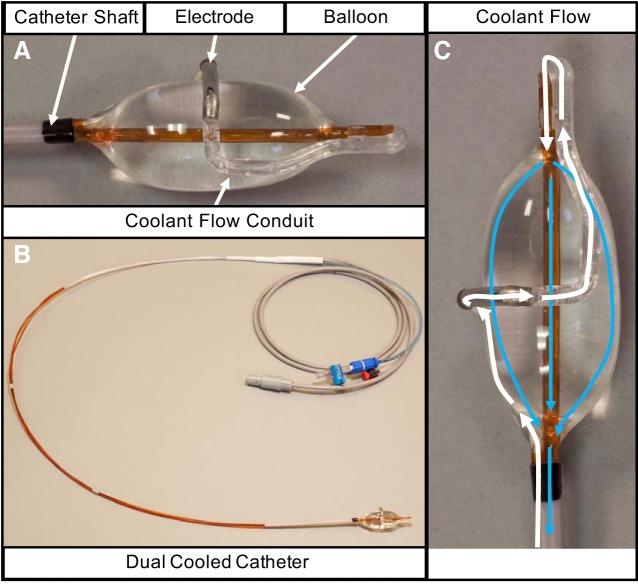
Depiction of the experimental catheter with dual cooled design. *A*: distal end of the catheter consists of the catheter shaft connected in series to the coolant flow conduit. The coolant flow conduit is separated into two parts by the stainless-steel electrode. The distal segment of the coolant flow conduit makes a loop at the end of the catheter and connects to the balloon. The balloon is connected to the catheter shaft. *B*: during the targeted lung denervation procedure the dual cooled catheter is connected to a console consisting of a radiofrequency energy generator and coolant fluid pump. *C*: during inflation, coolant fluid flows into the conduit along the catheter shaft and into the coolant flow conduit (white arrows). The coolant flows through the electrode and out the coolant flow conduit into the balloon (blue arrows). The balloon inflates pushing the electrode up against the airway wall. Coolant flows out of the balloon into the catheter shaft and back to the console. Coolant flow through the electrode and balloon cools the airway wall in two locations during energy delivery. It cools the tissue interface between the electrode and the airway wall, and it cools the tissue immediately adjacent to the airway wall.

Targeted lung denervation (TLD), using a low profile ablation system employed via bronchoscopy, is a potential minimally invasive method of denervating the lungs of pulmonary vagal inputs. The TLD system (Fig. 1) (Nuvaira, Inc, Minneapolis, MN) delivers radiofrequency ablation targeting the peribronchial branches of the vagus nerve while employing a cooling balloon to protect the bronchial epithelium. The feasibility of TLD has been demonstrated in humans with preliminary clinical studies showing improvement in symptoms, pulmonary function, and exercise capacity in patients with COPD (21, 22, 36, 38). The present manuscript details a series of preclinical studies that demonstrate the physiologic and histopathologic effects of TLD, providing mechanistic insight into these clinical observations. The goal of these early animal studies was to determine the ability of the lung denervation system to disrupt vagal inputs to the lung and produce a physiologic response.

## MATERIALS AND METHODS

### Study Design

Eleven sheep and two dogs underwent the TLD procedure in both main bronchi using a lung denervation system. All studies were conducted under the guidance of an Institutional Animal Care and Use Committee in accordance with the standard operating procedure of the study facility and in compliance with the Animal Welfare Act of 1966 and the ARRIVE guidelines.

Three sheep were evaluated for acute changes in pulmonary resistance before and following the TLD procedure. Two sheep and two dogs were tested for the presence or absence of the Hering-Breuer reflex (HBR) before, acutely following, and seven days following the TLD procedure. Six sheep were survived for 30 days post-TLD and underwent histologic evaluation for pulmonary nerve disruption and effects on peribronchial structures.

#### Targeted lung denervation procedure.

Procedures were performed under general anesthesia using intravenous and/or inhaled anesthetics following a 24-h fast. Animals were intubated with a large endotracheal tube (10 mm internal diameter). A flexible fiber-optic bronchoscope was passed, and the right and left mainstem bronchi were visualized and inspected. The ablation catheter was passed through the endotracheal tube and alongside the bronchoscope. The catheter tip was positioned in the mainstem bronchi, and single activations of radiofrequency energy were delivered to eight different rotational positions. Once activations were completed, the ablation catheter was directed into the contralateral bronchus, which was then treated in similar fashion. Ablation was performed at 20 W at all locations except the posterior octants on the left where power was reduced to 15 W due to potential proximity to the esophagus. One animal undergoing HBR analysis was treated at the lower power 15 W at all locations. Bronchoscopic evaluation of airway wall integrity was performed at the end of the procedure.

**Fig. 2.**
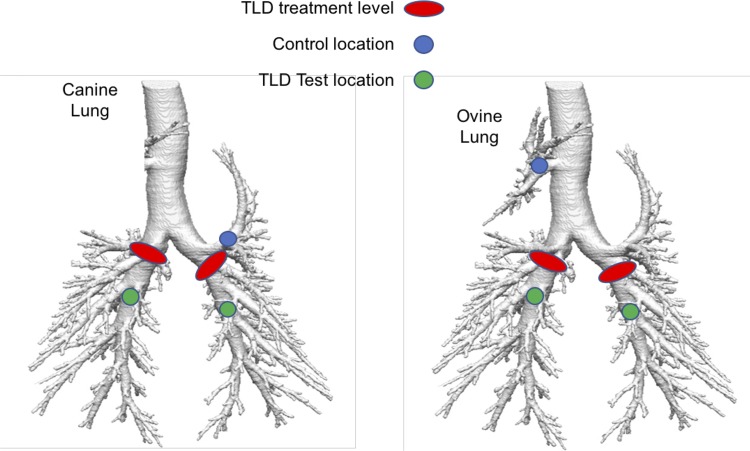
Targeted lung denervation (TLD) treatment sites in the ovine and canine models. Sites of ablation (red oval) in the canine and ovine models are shown. Locations distal to the treatment site (green circle) were used to test for extinction of the Hering-Breuer reflex after ablation. Early branching occurs of the left upper lobe on the canine lung and right upper lobe of the ovine lung. Thus the TLD treatment site was located distally to these lobes, which are thus, not denervated and serve as control locations (blue circle).

The sites of TLD treatment are depicted in Fig. 2. Early branching of the left upper lobe in the proximal left mainstem bronchi occurs in the canine lung. In the ovine lung, the right upper lobe originates directly from the trachea proximal to the left and right mainstem bronchi altogether. Thus TLD, which was performed in the right and left mainstem bronchi distal to these lobes, would not be expected to alter their innervation. For purposes of histopathologic evaluation and physiologic stimulation for the HBR, these untreated upper lobes were used as control locations.

#### Assessment of changes in airway resistance.

Resistance to pulmonary airflow was monitored in three sheep before and following TLD therapy to evaluate physiologic changes in airflow resistance. Each animal underwent a series of pulmonary resistance measurements using a custom-built forced oscillometry system (15). A 5-Hz pressure wave was generated by a custom speaker box and superimposed on the animals’ controlled breathing. The animals’ breathing was controlled by a ventilator set to 10–15 beats/min, tidal volume (VT) 0.5 L, inspiratory pressure (P_insp_) = 10 cm H_2_O, and positive end-expiratory pressure = 5 cm H_2_O. Airflow was measured by a calibrated, heated pneumotachograph, and pressure was measured by a calibrated pressure transducer. Pneumotachograph and pressure transducer output were displayed on an oscilloscope, the 5-Hz component isolated, and airway impedance calculated with airflow and pressure. A dose of 0.1 to 0.2 mg/kg of intravenous atropine was given to the sheep, and airway resistance was measured every 10 min until a maximum decrease in pulmonary resistance was measured. Two subsequent measures of pulmonary resistance were recorded at 10 min and 25 min following the peak measurement. After return to baseline airway resistance, TLD was performed. Airflow resistance was measured again at 30, 60, 90, and 120 min following the TLD procedure. The animals were killed following the final post-TLD measurement.

#### Assessment of the HBR.

The HBR was induced in lightly anesthetized and voluntary breathing sheep (two) and dogs (two) using a triple lumen, balloon-tipped catheter. The balloon-tipped catheter was placed in the lung in multiple locations to assess the functionality of the HBR before and following TLD (Fig. 2). Lobes located proximal to the treatment site were used as HBR controls, because these sections of lungs remained innervated following the TLD procedure. Locations in the distal left and right mainstem bronchi beyond the site of TLD therapy were used to evaluate sensory denervation and disruption of the HBR following the TLD procedure. To assess the presence or absence of the reflex, flow in and out of the endotracheal tube was measured while the triple-lumen balloon was inflated, and the lung distal to the balloon was pressurized to stimulate the HBR. Cessation of respiratory flow during balloon inflation and lung pressurization represents a positive HBR and intact sensory innervation of the occluded and pressurized portion of the lung. Normal breathing during balloon inflation and lung pressurization represents disruption of the HBR and loss of innervation to the isolated section of lung.

### In-Life Observations and Evaluation of Pathology

The remaining six sheep survived for 30 days post-TLD and were evaluated daily following TLD for clinical symptoms. Particular attention was given to the animals’ appetite and fecal and urinary production. If a health problem developed, the animal received appropriate tests or medical therapy as deemed necessary by a veterinarian.

#### Gross evaluation.

Follow-up bronchoscopy was performed at 30 days, and the animals were killed. The chest cavity was opened and the heart, pericardium, aorta, esophagus, mediastinum, and lungs were evaluated grossly. Any observed gross lesions were trimmed for processing and evaluation. The lungs and bronchi were inflated with 10% neutral buffered formalin at 20 to 25 cm water pressure to allow fixation near their normal expanded state. Following four days of fixation, dissection was performed to allow gross visualization of the mucosal surface of each bronchus, which was then trimmed into 9–12 sequential cross sections centered around the treatment site ~5 mm in thickness. Dissection of the esophagus was performed with particular attention to the right and left main periesophageal vagal trunks. Sections of right and left vagal trunks and adjacent esophageal wall at and distal to the treatment sites were trimmed.

#### Histologic assessment of bronchial and peribronchial structures.

All trimmed tissues were then processed through graded alcohols, cleared in xylene, embedded in paraffin, sectioned at 5 μ and stained with hematoxylin-eosin for light microscopic evaluation. The sections were then analyzed by a blinded certified veterinary pathologist as described below to quantify the histologic effects of the TLD procedure.

**Fig. 3.**
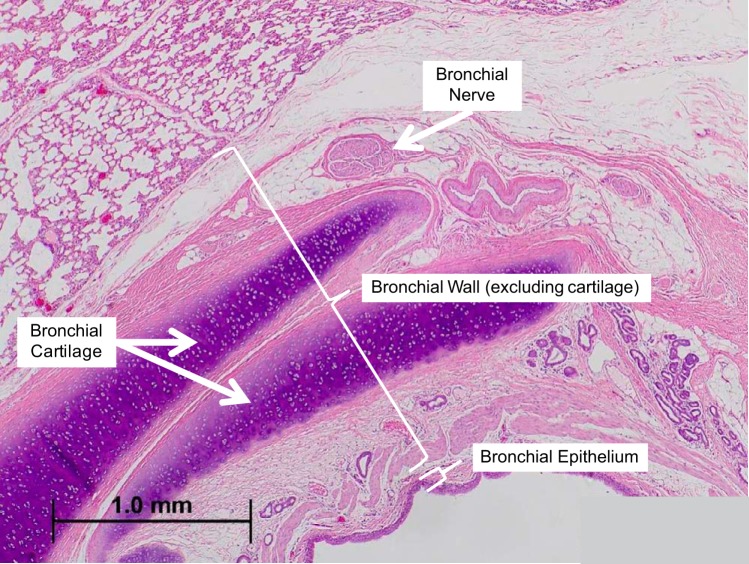
Grouping of bronchial structures for semiquantitative analysis. The tissue that makes up and immediately surrounds the airway was divided into four separate structures for analysis. The bronchial epithelium was scored as a single tissue. The bronchial lamina propria, smooth muscle, submucosa, and adventitial layer between airway and alveolar tissue make up the tissue evaluated as the bronchial wall. The bronchial cartilage was scored independent of the bronchial wall. Bronchial nerves were scored using immunohistochemical analysis and a nerve located in the adventitia that immediately surrounds the airway is shown (scale bar is 1 mm).

The bronchial epithelium, bronchial wall (defined as the lamina propria, smooth muscle, submucosa, and adventitial layer between airway and alveolar tissue), bronchial cartilage, surrounding alveolar parenchyma, surrounding blood vessels (the pulmonary arteries and veins), and esophagus were assessed histologically for the presence of necrosis, acute and chronic inflammation, and fibrosis (Fig. 3). All anatomic structures were evaluated from cross sections taken at the treatment site, and at sites 1 cm proximal and 1 cm distal to the treatment site. Potential tissue effects were graded as either not present, minimal, mild, moderate, or severe. In addition, epithelium was also assessed for hyperplasia and metaplasia, alveolar parenchyma for airway ectasia, and blood vessels for neointimal proliferation and thrombosis.

The percentage of the airway circumference at the treatment site at the time of airway harvest (one or more sections) that was lined by viable epithelium was determined by visual estimation. This assessment was defined as the epithelial integrity of the airway following treatment.

#### Immunohistochemical assessment of lung denervation.

Immunohistochemical staining was performed on select airway cross sections at each treatment site as well as proximal and distal to the treatment site. A commercial monoclonal antibody cocktail called pan-neuronal marker (pNM) (1:200 dilution; Millipore Corporation, Billerica, MA) was used to identify all axonal subtypes (i.e., motor and sensory axons) running along the airway. Axonal staining using the pNM antibody was validated in a two-step process. Staining of sheep sciatic nerve with the pMN marker was combined with known histomorphology of axonal/neuronal structures to validate and optimize staining parameters of this antibody in the sheep model. The antibody was further optimized for staining of sheep lung tissue by staining main bronchial tissue cross sections with a systematic series of staining parameters that emphasized reduction of staining in nonneuronal tissue subtypes.

Axon staining intensity within individual nerve fascicles of 50 μ or greater in diameter was scored from 0 to 10 using a semiquantitative scale based on the decile of fascicle staining, where 0 = no staining of any axon fibers and 10 = staining of > 90% of the fascicle. The score of stained tissue sections proximal to the site of treatment were used as controls of normal staining and were compared with the score of stained tissue sections at and distal to the site of TLD treatment.

All analysis of immunohistochemical staining was performed by a certified veterinary pathologist blinded to the experimental groups.

### Statistical Analysis

All data are reported as mean ± standard error of the mean except where otherwise specified. One-way analysis of variance (ANOVA) was performed to reveal differences between group means. If differences were observed, Tukey’s test was used to determine pairwise differences between group means. *P* < 0.05 was considered significant.

## RESULTS

### Assessment of Airway Resistance Following TLD

**Fig. 4.**
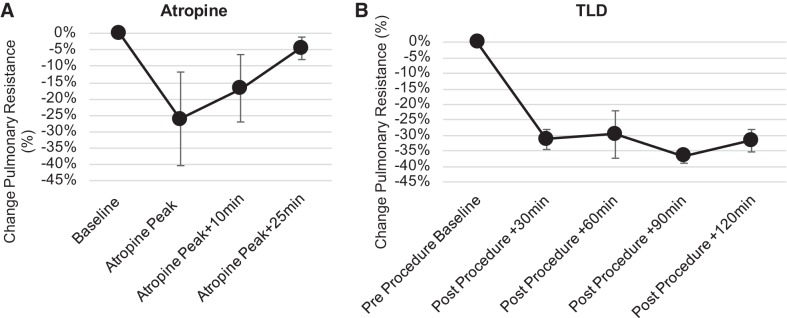
Assessment of changes in airway resistance with targeted lung denervation (TLD). Airway resistance was measured using a forced oscillometry system in three sheep before and after atropine and TLD. Changes in airway resistance are expressed as percent change from baseline. *A*: peak reduction in airway resistance of roughly 25% was seen following administration of x mg/kg of atropine, and returned to baseline within 25 min after peak effect. After return to baseline, changes in airway resistance were reassessed before and after TLD. *B*: a 30% reduction in airway resistance was seen postdenervation, which persisted for at least 2 h. Data are expressed as means and error bars as mean ± SE.

The atropine control results are shown in Fig. 4*A* and demonstrate a time-dependent bronchodilator effect of atropine that at peak produces on average 26 ± 14% reduction in pulmonary resistance in the sheep. The bronchodilator effect resolves 25 min following the peak effect of the drug. TLD produces a similar bronchodilator effect in the sheep resulting in a 30 ± 3% reduction in pulmonary resistance in the sheep Fig. 4*B*. In contrast to the atropine, the bronchodilator effect of TLD is sustained for the remainder of the study.

### Stimulation of HBR Following TLD

**Fig. 5.**
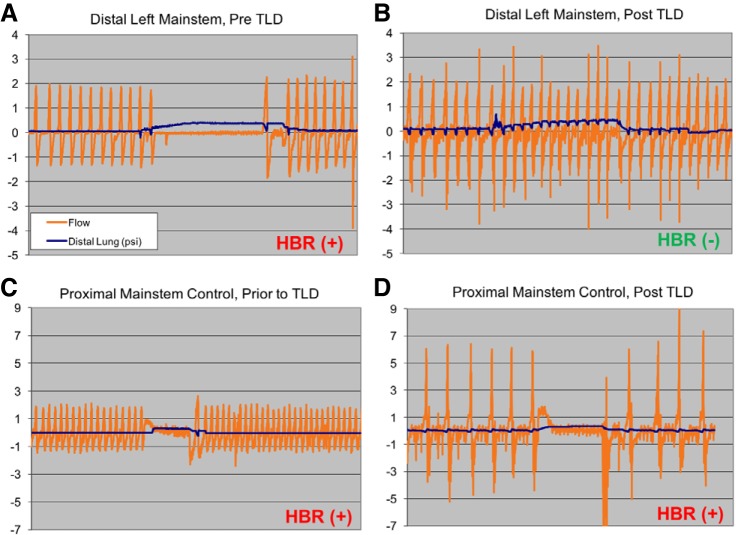
Suppression of Hering-Breuer reflex (HBR) at positions distal to ablation site HBR was assessed at points proximal and distal to the ablation site, before and after ablation in a canine model. Representative tracings in the left lung distal to the ablation site, before (*A*) and after (*B*) ablation are shown. As pressure in the distal lung (blue tracing) is increased by insufflating a balloon, respirations (air flow represented by orange tracing) are seen to be suppressed by the intact HBR before ablation (*A*). After ablation, the HBR is abolished due to removal of the vagal afferent limb of the reflex (*B*). C and *D*: the HBR is assessed before and after ablation at a site proximal to ablation, which serves as a control. The HBR remains intact. TLD: targeted lung denervation.

Stimulation of the Hering-Breuer stretch reflex was used to assess sensory innervation of TLD-treated lungs of sheep and dogs. Fig. 5 shows representative breathing traces recorded in the TLD treated sheep and dogs. Prior to TLD, pressurization of the lung stimulates the HBR and caused cessation of normal breathing (Fig. 5*A*). Following TLD, in the denervated sections of the lungs, over pressurization of the lung failed to alter normal breathing patterns (Fig. 5*B*). Prior to and following TLD, the control regions of the lung maintained the HBR (Fig. 5, *C* and D). Table 1 lists the outcomes of each HBR assessment in the four animals. With the exception of the animal treated at low power, the HBR was abolished or attenuated by the TLD acutely (0 day) and at seven days postprocedure.

**Table 1.** Response to Hering-Breuer reflex

					Hering-Breuer Reflex** (+/**−**)**		
Animal	Model	Survival, days	Lung	Power, W	*Day 0* (Pre)	*Day 0* (Post)	*Day 7* (Post)
1	Canine	0	R	20	+	−	NA
			L	20	+	−	NA
			C	NA	+	+	NA
2	Canine	7	R	20	+	−	−
			L	20	+	−	−
			C	NA	+	+	+
3	Ovine	0	R	15	+	+	NA
			L	15	+	+	NA
			C	NA	+	+	NA
4	Ovine	7	R	20	+	−	+/−
			L	20	+	−	−
			C	NA	+	+	+

R, right lung; L, left lung; C, control lobe; +, positive observation of Hering-Breuer reflex (HBR); −, absence of the HBR; Pre, before targeted lung denervation (TLD) therapy; Post, following TLD therapy; NA, not applicable.

### In-Life Observations and Measurements Following TLD

Overall, there were no clinically evident complications from the TLD procedure. All sheep were deemed healthy by an onsite veterinarian throughout the entire study and survived until their scheduled necropsy. No animals had any clinical events during the 30 days of follow-up, and all maintained normal appetites and weight over the course of the study. Blood cell counts, chemistries, and arterial blood gases showed no significant abnormalities.

### Bronchoscopic Assessment of Airway Wal

**Fig. 6.**
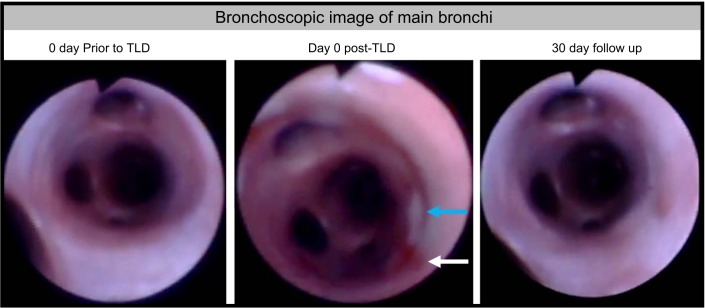
Bronchoscopic images of treated airway. Bronchoscopic images were taken on *day 0* pre- and post-TLD and during the 30-day follow-up. Immediately post-TLD areas of blanching (blue arrow) and hyperemia (white arrow) were seen at some of the treated locations. At 30 days, treatment sites were largely indistinguishable from baseline. TLD: targeted lung denervation.

Mild airway discoloration was seen in three of the airways acutely, immediately following TLD therapy. At 30 days, all main bronchi appeared essentially normal with some instances of mild hyperemia at follow up (Fig. 6). There was an instance of mild stenosis of a side branch airway in one animal and an instance of scarring in the side branch airway of another animal. In both instances, it was noted acutely that the electrode was positioned in the orifice of the affected side branch airway.

### Gross Effect of TLD

No significant gross lesions were identified in the lungs of any of the animals. Thirty days following the TLD procedure, fibrotic replacement of lung parenchyma immediately adjacent to the treated airways was observed at essentially all treatment sites but was of minor extent, generally involving a sleeve of lung parenchyma only 1–2 mm thick immediately adjacent to the treatment site.

No serious effects in the major pulmonary arteries or veins were seen. There were no gross treatment-associated effects identified in the right and left main vagal branches traveling along the esophagus distal to the treatment site.

No serious effects were identified grossly in the esophagus at 30 days. Gross inspections of the heart, pericardium, aorta, and mediastinum revealed no treatment related effects.

### Histologic Effect of TLD

#### Immunohistochemical assessment of lung denervation.

**Fig. 7.**
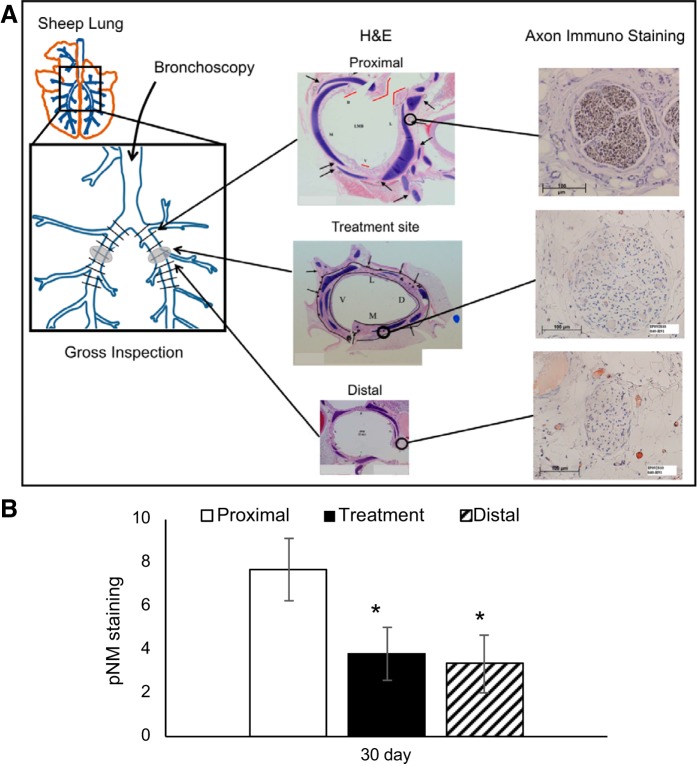
Immunohistochemical analysis of the nerve staining in the sheep airway. *A*: immunohistochemical staining was performed on select airway cross sections at each treatment site, as well as proximal and distal to the treatment site in six sheep (12 airways). A commercial monoclonal antibody cocktail called Pan Neuronal Marker (pNM) was used to identify all axons. A semiquantitative assessment was developed to evaluate the extent of axonal staining in nerve fascicles surrounding the airways. Axon staining intensity within individual nerve fascicles was scored from 0 to 10. The figure presents representative images of nerve fascicle scores proximal, at, and distal to the site of treatment. The score of stained tissue sections proximal to the site of treatment were used as controls of normal staining and were compared with the score of stained tissue sections at and distal to the site of targeted lung denervation (TLD) treatment site. *B*: semiquantitative analysis of pNM staining in TLD treated nerves. At 30 days post-TLD, a significant decrease in bronchial nerve expression of pNM demonstrates significant disruption of bronchial axons following TLD. Bars are averages, error bars are standard error of the mean. *P < 0.05 vs. proximal control. H&E, hematoxylin-eosin.

Immunohistochemical analysis using a pan neuronal antibody cocktail was used to quantify the effect of TLD therapy on motor and sensory axons located within the targeted bronchial peripheral nerves. The antibody cocktail, in part, identifies neurofilament expression in both motor and sensory axons. Neurofilament is a cytoskeletal protein that provides the structural support for axons and is unique to neuronal cells (37). Following injury, the expression of neurofilament is maintained in the proximal portion of the injured axon and is lost at the site of injury and in the distal component of the axon. At 30 days following TLD therapy there was a significant decrease in pNM expression at the site of treatment (3.8 ± 1.2) and distal (3.4 ± 1.3) to the treatment site when compared with proximal expression (7.7 ± 1.4) (Fig. 7).

### Histological Effects of TLD on Bronchial and Peribronchial Structures

#### General histologic effects.

**Fig. 8.**
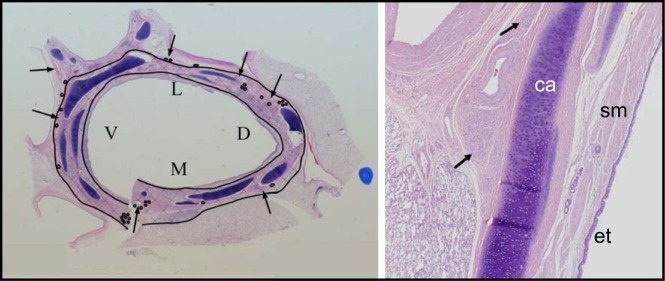
Histologic section of main bronchi targeted lung denervation (TLD) produces a zone of ablation within the airway wall that encapsulates the majority of airway nerve fascicles. *A*: representative low power (10 ×) histologic cross section of bronchi at the treatment site 30 days post-TLD. Hematoxylin-eosin staining was used to delineate tissue structures in the bronchi. Inner and outer black lines delineate the zone of ablation evident by dense fibrotic scarring in the tissue and highlighted by the cross-hatched region in the image. The region between the drawn inner black line and the airway surface is a zone of epithelial protection provided by the cooled balloon and electrode of the catheter. Arrows indicate branches of the bronchial arteries and the circles indicate nerve fascicles. V, M. L, and D indicate the ventral, medial, dorsal, and lateral aspect of the airway respectively. *B*: high power (40 ×) view of one segment is shown. The fibrotic area characteristic of the treatment zone is depicted by arrows. Cartilage (ca), smooth muscle (sm), and bronchial epithelium (et) remain intact.

The TLD treatment effect consisted of a band of remodeled tissue in the outer layers of the bronchial wall and the surrounding adventitia (Fig. 8, cross-hatched region). This band of remodeled tissue consisted of well-organized fibroplasia extending outward from the submucosa through the full thickness of the airway wall. It often entrapped, and occasionally obliterated, normal structures including nerves, submucosal glands, bronchial smooth muscle, small blood vessels and bronchial cartilage. Moderate-to-severe fibrosis was consistently seen through the adventitial layers of the bronchi (where branches of the vagus nerve are located).

The band of fibrosis and remodeled tissue was typically separated from the lumen of the airway by a protected layer, which often included normal bronchial epithelium, lamina propria, and some smooth muscle tissue (Fig. 8, inner line).

#### Bronchial epithelium.

The dual-cooled catheter design provides surface protection to the bronchial epithelium via coolant circulating through both the catheter electrode and balloon. Histologically a layer of protected tissue extending from the surface of the airway in to the wall to the submucosal layer (Fig. 8, inner line) was seen, which exhibited only mild inflammatory changes without any evidence of fibrosis or necrosis at 30 days. The bronchial epithelium immediately proximal and distal to the treatment site was absent of virtually all tissue effects.

#### Bronchial wall.

Durable denervation of the lung requires disruption of motor axons and formation of fibrosis within bronchial nerve branches located in the adventitial layers surrounding the bronchial wall to prevent reinnervation. At 30 days, the outer layer of the bronchial wall was largely replaced by fibrosis at the depth of these branches. Moderate fibrosis was consistently seen associated with only mild chronic and minimal acute inflammation. Tissue necrosis was largely absent. Minimal levels of chronic inflammation in the bronchial wall are considered normal in untreated sheep bronchi and were observed proximal and distal to the treatment site.

#### Bronchial cartilage.

The bronchial cartilage is located within the outer layers of the bronchial wall but was scored as a separate tissue type because of its distinctive anatomy in the sheep and its unique response to TLD therapy. The cartilage in sheep bronchi consists of overlapping plates that are always situated directly below the TLD electrode. Necrosis of this cartilage at the treatment site was seen at 30 days. Chronic and acute inflammation was largely absent. Cartilage proximal and distal to the treatment site displayed no pathological effects of treatment.

#### Alveolar parenchyma.

In some locations surrounding the TLD treatment site, alveolar parenchyma is situated within close proximity to the outer edge of the bronchial wall. Mild-to-moderate fibrosis, airway ectasia, hyperplasia, metaplasia, and chronic inflammation were all observed at 30 days in the small portion of alveolar tissues surrounding the treatment site. Minimal acute inflammation was present, and necrosis was largely absent. Macrophages and hemosiderophages (i.e., macrophages containing hemosiderin, a breakdown pigment of erythrocytes) were a component of the cells that made up the chronic inflammation in the alveoli.

The alveolar tissue immediately proximal and distal to the treatment site was essentially normal. There was minimal chronic inflammation, which is a common finding in sheep lung and likely not a result of TLD therapy. All other findings were absent.

The average depth of parenchyma affected by TLD therapy was < 2.2 mm. Considering this depth, the average length of alveolar parenchyma affected, and the radius of the treated airways, the maximum total volume of alveolar parenchyma affected by TLD was calculated to be ~8 × 10^−4^ l or < 0.05% of the lungs.

#### Bronchial blood vessels and the esophagus.

The bronchial blood vessels and esophagus were also analyzed for TLD-related tissue effects. There were no observations of histologic changes in major blood vessels (the pulmonary arteries and veins) at the treatment site.

Histologic changes in the esophagus were limited to two animals at the 30-day time point. These animals showed regional areas of fibrosis and mild inflammation in the outer smooth muscle layer, likely a result of treatment. Minimal chronic inflammatory changes were also noted in the muscularis of other animals, which was considered unrelated to the treatment and likely associated with a common parasite (Sarcocystis) found in ruminants and was specifically identified in some of the affected animals.

#### Periesophageal branches of the vagus nerve.

The analysis of pNM expression was also performed in the main trunks of the vagus nerve running along the esophagus distal to the treatment location. All examined vagus nerve trunks demonstrated normal pNM staining (6 of 6) showing that the main trunks of the vagus nerve were unaffected by TLD treatment.

## DISCUSSION

The primary target of TLD is the preganglionic axons of motor neurons contained within the pulmonary branches of the vagus. TLD therapy would ideally disrupt these motor axons and also produce sufficient fibrosis to prevent their ability to regenerate. TLD altered the pulmonary physiology of the treated animals, evident by a reduction in airway resistance and abolition of the vagal sensory mediated HBR. Thirty-day follow-up demonstrated a ring of fibroplasia surrounding the airway at the depth of the vagus nerve branches that engulfed and tended to replace these nerve branches. The present study suggests that TLD produces an adequate lesion depth to reliably denervate pulmonary branches of the vagus and alter lung physiology.

### Effect on Airway Resistance and HBR

In the absence of obstructive airway disease, the efferent innervation of the lung provides a base level of bronchial smooth muscle tone. TLD resulted in a 30% reduction in resistance to air flow in the lung. The reduction is consistent with changes in pulmonary resistance reported in the literature evaluating the effect of vagotomy on airway mechanics (5, 8, 9, 18, 20, 27). The change in pulmonary resistance suggests a change in airway caliber which is likely due to a change in cholinergic tone. The similar reduction in pulmonary resistance observed through the action of the atropine control provides further evidence that the TLD-induced reduction was a result of efferent denervation of the lung.

In addition to efferent motor input, an extensive network of vagal sensory fibers also innervates the lung via vagal nerve branches. These sensory fibers innervate several sets of bronchopulmanry sensory receptors that are specialized for detecting changes in chemical, mechanical, or thermal stimuli. The bipolar vagal sensory neurons are located near the spinal cord in the nodose and jugular ganglia, and participate actively in reflex events that regulate breathing and bronchoconstriction/dilatation (17, 24, 41). Pulmonary slowly adapting stretch receptors respond to changes in lung volume and when activated by sustained lung inflation, reflexively inhibit the onset of the subsequent inspiration, known as HBR inflation (40).

Following TLD, the HBR was abolished in the majority of the treated animals, consistent with previously observed effects of vagotomy (17). The exceptions were one animal treated at a lower radiofrequency (RF) power and another animal who regained some of the reflex at seven days post-TLD. Lower power used to treat this animal may not have produced a lesion of sufficient depth to capture all of the sensory axons surrounding the airway. The spared sensory axons were likely responsible for the retention of the HBR. A power dose-related response has been observed with TLD during clinical investigations (36). In the other animal, the reflex didn’t return completely but did serve to diminish the rate of breathing during balloon inflation. Sensory axons have been shown to functionally regenerate more efficiently than motor axons (34) and could account for this animal’s partial return of the HBR. The combined loss of the HBR and reduction of pulmonary resistance following TLD are evidence of neural modulation of the lung.

### Effect on Bronchial and Peribronchial Structures

The dual-cooled catheter design allows protection of the airway surface while delivering heat at depth to the airway wall. Damage to the airway surface from heat energy has been shown to result in a healing process that ends with airway stenosis (7, 11, 19). Specifically, circumferential radiofrequency ablation at six equidistant points in the airway lumen has been used to create a model of tracheal stenosis. By three weeks in this tracheal stenosis model, the trachea of ablated animals presented with between 84 and 94% stenosis of the trachea (26). In the absence of cooling, it would be expected that a similar amount of stenosis would result following the TLD procedure. The lack of any substantial stenosis in TLD-treated airways from this study demonstrates the impact and importance of surface cooling employed by this ablation system.

The ablation electrode is designed to sit between discrete rings of cartilage within human main bronchi, thus minimizing the effect of TLD on cartilage in humans. Ovine bronchi, in the treatment area, are supported by overlapping plates of cartilage that lie directly beneath the ablation site and are thus subjected to a high intensity of delivered energy. This sheep-specific anatomy provides a very sensitive model of cartilage tissue effects that we would not expect to see in humans. Cartilage necrosis and subsequent fibrosis were seen in all the treatment zones. This injury to cartilage did not result in any obvious gross changes in airway architecture, and no evidence for malacia was seen in any airways.

As the depth of ablation often exceeds the thickness of the bronchial wall, collateral damage to nearby peribronchial structures may potentially occur. Most often, the ablation sites within the mainstem bronchi are directly contiguous to pulmonary parenchyma, relatively large branches of the pulmonary arteries and veins, and mediastinal fat. However, the posterior aspect of the bronchi (especially on the left) may be in close proximity to the esophagus, which is motile and has a variable course. The heart and aorta may also be adjacent to treatment sites.

TLD treatment effects were often seen in alveolar parenchyma immediately adjacent to the bronchi. However, only a trivial volume (< 0.05%) of lung tissue was affected, which would not be expected to have any significant physiologic effect.

TLD treatment sites were also often contiguous to branches of the pulmonary arteries and veins. There were no observed histologic changes in these major blood vessels. Large blood vessels would be expected to be protected from thermal injury by the cooling effect of blood flow (12, 28, 35).

The esophagus is sometimes located in close proximity to posterior ablation sites, particularly at more proximal ablation sites on the left. Significant complications have been reported during left atrial ablation relating to either damage to the esophagus directly or to branches of the periesophageal vagi resulting in atriesophageal fistulae and gastroparesis, respectively (1, 4, 23, 25, 26, 32). Thus, care was taken to reduce power (to 15 W) in the posterior ablation octants on the left side to avoid potential complications. With this strategy, effects on the esophagus were minimal with only two animals showing mild fibrosis in the outer smooth muscle layer of the esophagus at 30 days. No histologic effects were seen in the periesophageal branches of the vagus, nor were there any clinical changes in any animal’s appetite or weight.

### Potential Implications for Treatment in COPD

Preliminary studies in man have shown that TLD is feasible and safe to perform via rigid bronchoscopy in patients with COPD (36, 38). TLD conferred additional benefit to inhaled anticholinergics in these studies resulting in improvement in symptoms, exercise capacity, and pulmonary function in patients with moderate to severe COPD. TLD may thus become an important treatment modality in patients with obstructive lung disease who remain symptomatic, despite optimal pharmacologic therapy. Other effects on pulmonary physiology mediated by the parasympathetic nervous system, such as mucus production, cough, reflex bronchoscontriction and inflammation, will need to be assessed in clinical studies. In addition to the effects of vagal denervation, potential procedural complications of ablation include collateral damage to structures in proximity to the intended target. While particular care must be taken when performing ablation to tissue close to the esophagus to prevent potential bronchesophageal fistulae formation or damage to branches of the periesophageal vagus nerve, this study shows fairly minimal damage to the bronchial epithelium, cartilage, vasculature, and other peribronchial structures.

### Conclusion

TLD performed in both right and left bronchi in these animal models reduced pulmonary airway resistance, abolished the HBR, and consistenty resulted in bronchial wall fibrosis to a depth which engulfed nearly all pulmonary branches of the vagus nerve. Immunohistochemical analysis demonstrated a marked decrease in neurofilament expression distal to the treatment site consistent with effective denervation. The novel dual-cooled catheter design provided efficient protection of bronchial epithelium and caused minimal collateral damage to other peribronchial structures. Thus TLD therapy, performed at similar power output settings to those used in preliminary studies in humans (36, 38), appears to result in safe and effective pulmonary vagal denervation. Vagal denervation is thus the likely mechanism by which patients have derived clinical benefit in these studies. The use of TLD in the treatment of chronic lung disease where parasympathetic activity plays an important pathophysiologic role warrants further investigation.

## DISCLOSURES

Dr. Hummel is a paid consultant, equity holder, and member of the Scientific Advisory Board for Nuvaira, Inc. Dr. Mayse is the founder and present Chief Technology Officer of Nuvaira, Inc. Steve Dimmer is the former Chief Executive Officer of Innovative Pulmonary Solutions (previous name of Nuvaira) and a significant equity holder in Nuvaira, Inc. Dr. Johnson is a paid employee of Nuvaira, Inc.

## AUTHOR CONTRIBUTIONS

J.P.H., M.L.M., S.D., and P.J.J. performed experiments; J.P.H., M.L.M., S.D., and P.J.J. analyzed data; J.P.H., M.L.M., S.D., and P.J.J. interpreted results of experiments; J.P.H., M.L.M., and P.J.J. drafted the manuscript; J.P.H., M.L.M., S.D., and P.J.J. edited and revised the manuscript; J.P.H., M.L.M., S.D., and P.J.J. approved the final version of manuscript; M.L.M. and P.J.J. prepared figures.
